# The terminal nerve plays a prominent role in GnRH-1 neuronal migration independent from proper olfactory and vomeronasal connections to the olfactory bulbs

**DOI:** 10.1242/bio.029074

**Published:** 2017-10-02

**Authors:** Ed Zandro M. Taroc, Aparna Prasad, Jennifer M. Lin, Paolo E. Forni

**Affiliations:** Department of Biological Sciences, University at Albany, Albany, NY 12222, USA

**Keywords:** GnRH-1 neurons, Kalmann Syndrome, Olfactory bulbs, Olfactory neurons, Vomeronasal organ

## Abstract

Gonadotropin-releasing hormone-1 (GnRH-1) neurons (GnRH-1 ns) migrate from the developing olfactory pit into the hypothalamus during embryonic development. Migration of the GnRH-1 neurons is required for mammalian reproduction as these cells control release of gonadotropins from the anterior pituitary gland. Disturbances in GnRH-1 ns migration, GnRH-1 synthesis, secretion or signaling lead to varying degrees of hypogonadotropic hypogonadism (HH), which impairs pubertal onset and fertility. HH associated with congenital olfactory defects is clinically defined as Kallmann Syndrome (KS). The association of olfactory defects with HH in KS suggested a potential direct relationship between defective olfactory axonal routing, lack of olfactory bulbs (OBs) and aberrant GnRH-1 ns migration. However, it has never been experimentally proven that the formation of axonal connections of the olfactory/vomeronasal neurons to their functional targets are necessary for the migration of GnRH-1 ns to the hypothalamus. Loss-of-function of the *Arx-1* homeobox gene leads to the lack of proper formation of the OBs with abnormal axonal termination of olfactory sensory neurons ( Yoshihara et al., 2005). Our data prove that correct development of the OBs and axonal connection of the olfactory/vomeronasal sensory neurons to the forebrain are not required for GnRH-1 ns migration, and suggest that the terminal nerve, which forms the GnRH-1 migratory scaffold, follows different guidance cues and differs in gene expression from olfactory/vomeronasal sensory neurons.

## Abstract (precis)

**Summary:** Our work reveals that correct olfactory bulb development is not required for GnRH-1 neuronal migration. This study challenges the idea that GnRH-1 neuronal migration to the hypothalamus relies on correct routing of the olfactory and vomeronasal neurons and supports the existence of the TN in mammals.

## INTRODUCTION

Gonadotropin-releasing hormone-1 neurons (GnRH-1 ns) play a pivotal role in controlling the reproductive axis of vertebrates. In the adult, the GnRH-1 ns reside within the preoptic hypothalamic area (POA), where they control the hypothalamic–pituitary–gonadal hormonal axis (HPG axis), driving reproductive development and regulating reproductive hormones in adult life (Cattanach et al., 1977). During embryonic development, the GnRH-1 ns originate in the developing olfactory pit (OP), from which they migrate into the brain and eventually arrive at the hypothalamus (Schwanzel-Fukuda and Pfaff, 1989; Wray et al., 1989a,b). Disturbances either in this migration or in GnRH-1 synthesis, secretion, and signaling lead to hypogonadotropic hypogonadism (HH), which adversely affects normal sexual development, social interactions, fertility and propagation of the species (Burmeister et al., 2005; Maruska and Fernald, 2011; Schwanzel-Fukuda et al., 1989; Yin and Gore, 2006; Zhang et al., 2013). Around half of all patients affected by HH either have difficulty perceiving odors (hyposmia) or entirely lack the ability to smell altogether (anosmia) (Bianco and Kaiser, 2009; Mitchell et al., 2011). HH associated with congenital olfactory defects is clinically defined as Kallmann Syndrome (KS).

Developing GnRH-1 ns migrate along bundles of olfactory/vomeronasal (VN) and terminal nerve (TN) axons, which project from the nose to the olfactory bulb and POA, respectively. Whether these axons are collectively permissive for GnRH-1 neuronal migration from the pit to the brain, or whether a specific neuronal population provides guidance essential for successful migration has long been controversial. The prevailing idea is that GnRH-1 ns access the brain along the olfactory/vomeronasal (VN) sensory fibers (Boehm et al., 2015; Forni and Wray, 2015; Wray, 2010; Yoshida et al., 1995). This idea found further support in (1) evidence indicating that the GnRH-1 ns originate from the olfactory placode (Schwanzel-Fukuda et al., 1989; Schwanzel-Fukuda and Pfaff, 1989); (2) data showing that the GnRH-1 ns migrate to the hypothalamus in a neurophilic/axophilic fashion (Casoni et al., 2012; Yoshida et al., 1999); and (3) a loose correlation between HH and mutations in genes affecting innervation of the olfactory bulbs (Della Valle et al., 2013; Dodé et al., 2003, 2006; Forni and Wray, 2015; Kim et al., 2008; Miraoui et al., 2013; Pingault et al., 2013; Valdes-Socin et al., 2014).

Studies based on genetically modified animal models have described GnRH-1 ns migratory defects associated with an array of olfactory and vomeronasal sensory neuronal routing deficiencies, either alone or together with atypical formation of the olfactory bulbs (OBs) (Bergman et al., 2010; Boehm et al., 2015; Cariboni et al., 2015, 2012, 2011, 2007; Hanchate et al., 2012; Lettieri et al., 2016; Messina and Giacobini, 2013; Pingault et al., 2013). Unilateral and bilateral absence or reduction in the size of OBs, are common phenotypes in Kallmann patients carrying Kal1, CHARGE, trisomy 13 or trisomy 18 mutations, Prok2 or Prok-R2 mutations, and mutations affecting Fgf8 signaling (Dodé and Hardelin, 2010; Hardelin and Dodé, 2008; Ng et al., 2005; Pitteloud et al., 2007; Teixeira et al., 2010). Defective projections to the CNS and defective bulb formation have also been described after loss of function of *Dlx5*, *Fezf1*, *Klf7*, *Emx2* and *Lhx2* genes in mouse (Berghard et al., 2012; Chung et al., 2008; Hirata et al., 2006; Levi et al., 2003; Long et al., 2003; Yoshida et al., 1997; Yoshihara et al., 2005). Notably, these genes are expressed by olfactory placodal derivatives and brain.

Despite many correlations, direct experimental evidence proving that olfactory and vomeronasal connections to the OBs are necessary for GnRH-1 ns migration to the hypothalamus is lacking. Additionally, in families carrying mutations linked to KS, the two aberrant phenotypes, HH and anosmia, do not necessarily co-segregate (Balasubramanian et al., 2014; Frasnelli et al., 2007; Ghadami et al., 2004; Leopold et al., 1992; Pitteloud et al., 2006; Yousem et al., 1996).

Earlier researchers arrived at the conclusion that the GnRH-1 ns must reach the hypothalamus on a set of VN fibers (Wray et al., 1989a; Yoshida et al., 1995). However, other reports indicated that the GnRH-1 ns migrate to the hypothalamus along a distinct set of neurons that bundle with the olfactory and/or VN fibers but are not themselves olfactory/vomeronasal sensory neurons (OSNs/VSNs) (Schwanzel-Fukuda and Pfaff, 1989). Such neurons are believed to belong to the elusive cranial nerve cranial nerve-0 or the TN (Quintana-Urzainqui et al., 2014; Vilensky, 2012; Zhao et al., 2013).

Notably, whereas the VNO is absent or vestigial in primates, birds, amphibians, toothed whales and fish, the TN connecting the nose to the brain, exists in these species (Buhl and Oelschläger, 1986; Demski and Schwanzel-Fukuda, 1987; Dulac and Torello, 2003; Fuller and Burger, 1990; Mousley et al., 2006; Muske and Moore, 1988; Ridgway et al., 1987; Smith and Bhatnagar, 2000; Zhao et al., 2013).

*Arx-1 is* an X-linked homeobox gene related to the *Drosophila aristaless*. Arx-1 loss-of-function leads to a severe form of arhinencephaly together with abnormal axonal termination of olfactory sensory neurons (Yoshihara et al., 2005). Arx-1 is neither expressed by the olfactory neurons nor by GnRH-1 ns. This feature make the ARX-1 mutants an optimal model to test if proper development of the olfactory bulbs is necessary for GnRH-1 neuronal invasion of the brain. We have exploited Arx-1^null^ mutants together with a series of reporter mouse models to selectively track neurons in the developing nose. Our data suggest that proper olfactory bulb development and axonal connections of the olfactory and vomeronasal sensory neurons to the brain are not needed for GnRH-1 neuronal migration. In fact, the GnRH-1 ns and the putative TN appear to follow different guidance cues from those controlling the innervation of the OBs.

## RESULTS

### Arx-1 mutants lack proper olfactory bulb formation

As previously described in detail by Yoshihara and coworkers (Yoshihara et al., 2005), Arx-1^null^ mice develop a severe bulb aplasia/hypoplasia secondary to the defective proliferation, migration, and maturation of interneuron progenitors and precursors into the OB. Periglomerular cells and granule cells are two major types of GABAergic interneurons in the OB (Kiyokage et al., 2017; Kosaka et al., 1995; Mugnaini et al., 1984). Tyrosine hydroxylase (TH) is expressed by sets of periglomerular cells and cells of the molecular layer. Olfactory nerve input is required for the normal expression of TH in the main olfactory bulb (Ehrlich et al., 1990; Kawano and Margolis, 1982; Stone et al., 1990).

**Fig. 1.**
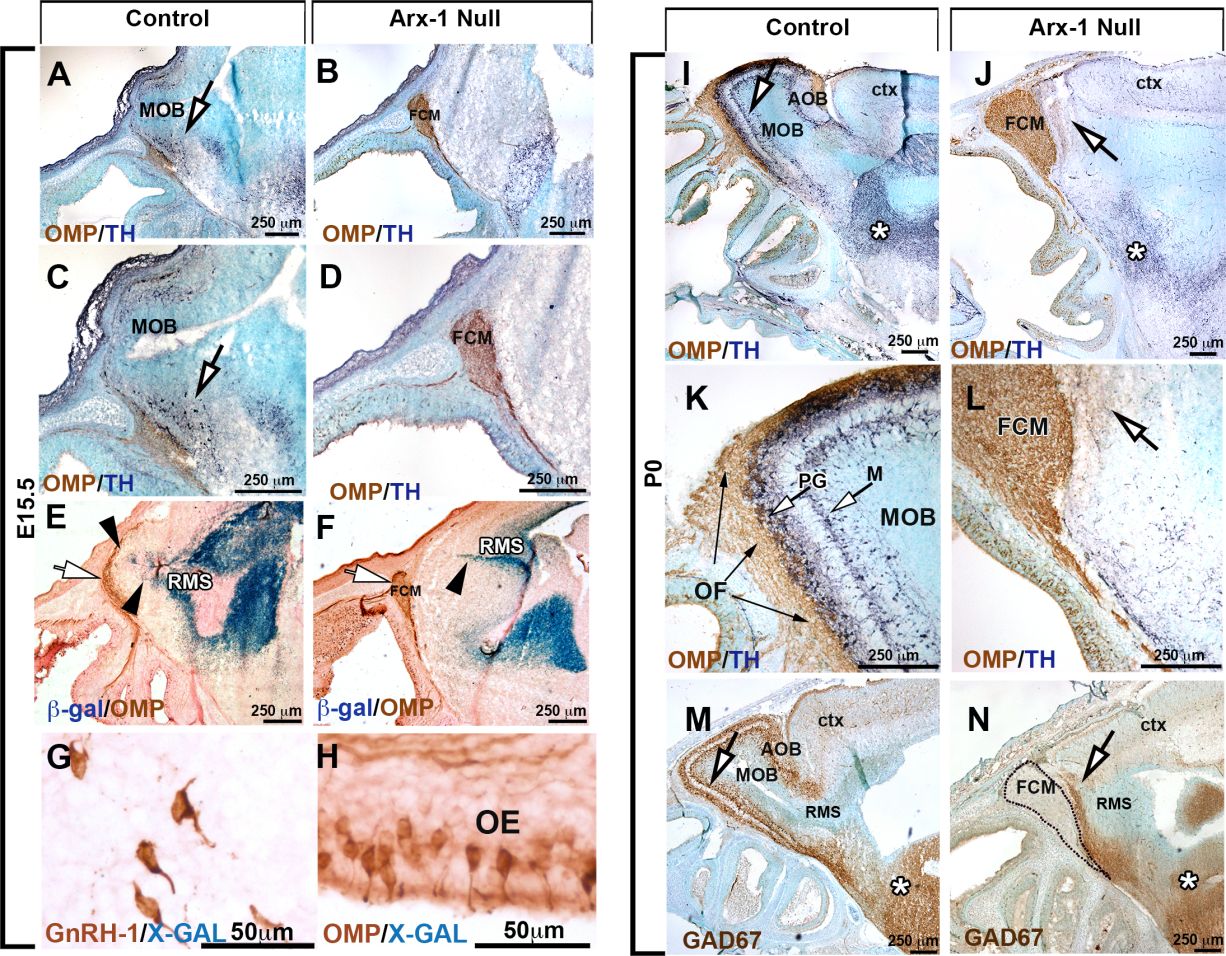
**The olfactory bulbs fail to develop properly in Arx-1^null^ mutant mice.** (A-D) Immunohistochemistry against OMP (brown) and TH (dark blue) on control (A,C) and Arx-1^null^ (B,D) mouse at E15.5 shows detectable TH cells in the developing OB of control animals (arrows) but not in the mutants, where the FCM stalls in front of the brain. OMP (brown)-positive fibers were found projecting and targeting to the MOB of control animals, while they collapsed proximal to the brain as part of the FCM in Arx-1 mutants. (E,F) OMP immunostaining and X-Gal staining on Arx-1 E15.5 Het control (E) and Arx-1^Null^ (F). In controls, Arx-1 was expressed in the rostral migratory stream and in cells invading the developing MOB (black arrowheads) innervated by the olfactory fibers (white arrows). In Arx-1^Null^ mutants the Arx-1+ cells fail to invade the developing MOB. (G,H) X-Gal staining on E15.5 Arx-1 Het. Arx-1 expression (blue) is not found in (G) GnRH-1 ns nor in (H) OMP+ olfactory neurons. (I,K,M) P0 Controls and (J,L,N) Arx-1^null^. (I-L) Immunohistochemistry against OMP (brown) and TH (dark blue) on control (I,L) and Arx-1^null^ mouse (J,L). In the control, TH-positive cells (white arrows) were found in the developing peri-glomerular (PG) and molecular layer (M); no TH+ cells were found in the Arx-1^null^ mutants (L,N) (arrows). (M,N) GAD67 immunostaining on P0 (M) control and (N) Arx-1^null^ mutant. In control animals, GAD67 immunoreactivity was detected in the MOB (arrow); in mutant mice, GAD67+ cells accumulated ventrally and at the rostral end of the RMS (arrow). Comparable GAD67 pattern of immunoreactivity between controls and Arx-1^null^ was detected in the striatum (asterisks).

Control mice immunostained for olfactory marker protein (OMP; to label olfactory and vomeronasal neurons and axons) and TH revealed the normal projections and active connections of olfactory/vomeronasal axons (Fig. 1A,C,I,K). In Arx-1^null^ animals, the olfactory fibers were found tangled in a large fibro-cellular mass (FCM) (Fig. 1B,D,J,L) and no TH immunoreactivity was found in the OBs (Fig. 1J,L).

Immunostaining against glutamic acid decarboxylase-67 (GAD67) (Carleton et al., 2003; Mugnaini et al., 1984) highlighted the well-organized GABAergic neurons in the OB of control animals (Fig. 1M). In Arx-1 mutants, most of the GAD67-positive neurons could not enter the OB and accumulated ventral and at the rostral end of the RMS (Fig. 1N). However, comparable TH and GAD67 immunoreactivity was found in the striatum of WT and Arx-1^null^ mutants (Fig. 1I,J,M,N, asterisks).

In the Arx-1^null^ mice, two exons have been replaced by the β-galactosidase gene (Kitamura et al., 2002). By performing X-Gal staining on Arx-1^null^ mutants and Arx-1^+/−^ controls (Fig. 1E,F), we confirmed the migratory defects of the interneuron progenitors, as reported by Yoshihara et al. (2005), together with the absence of Arx-1 expression in the olfactory epithelium and in GnRH-1 ns (Fig. 1G,H).

### Aberrant olfactory development does not affect GnRH-1 migration to the basal forebrain

**Fig. 2.**
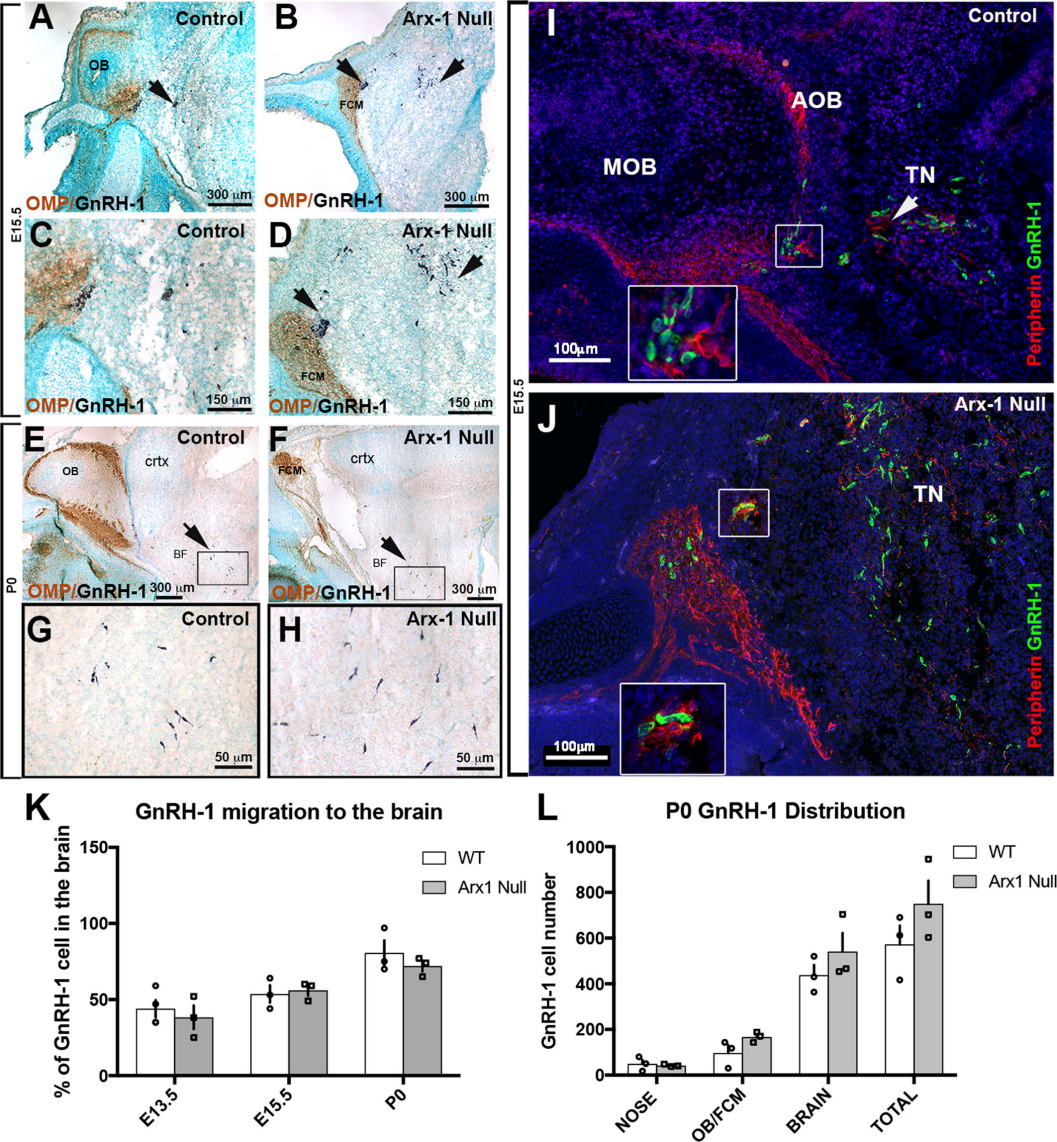
**GnRH-1 neuron migration is not altered in Arx-1^null^ mutants.** (A-H) Immunohistochemistry against OMP (brown) and GnRH-1 (blue) on control (A,C,E,G) and Arx-1^null^ (B,D,F,H) mouse at E15.5 (A-D) and P0 (E-H). In both controls and Arx-1^null^, the GnRH-1 ns (dark blue) migrate to the basal forebrain on OMP negative fibers. In Arx-1^null^ mutants (D) the GnRH-1 ns cross the axonal tangle of the FCM mass and migrate to the basal forebrain (F,H) as in control animals (E,G) (black arrows). (I,J) E15.5, double immunofluorescence against Peripherin and GnRH-1. The GnRH-1 ns migrate to the basal forebrain on Peripherin-positive fibers of the TN on both control and Arx-1^null^ mice (white arrows). Enlargements illustrate the TN separating from the olfactory fibers that project to the MOB in control (I) or collapsed in the FCM in Arx-1^null^ mutants (J). (K) At the three analyzed stages a similar percentage of GnRH-1 ns migrated to brain in controls and Arx-1^null^ mice. (L) Graphs of GnRH-1 ns cell counts at P0 in the nose, OB/FCM, brain and total. No statistical difference was seen in all areas between control and Arx-1^null^ (data are mean±s.e.m., unpaired student's *t*-test, *P*>0.05).

GnRH-1 ns start to invade the brain region around E12.5 and complete their migration in about 5 days. We analyzed Arx-1^null^ mice and wild-type controls at E13.5 and E15.5, which are stages in which the GnRH-1 ns are still migrating, and at the completion of embryonic development, P0. Double immunolabeling against OMP and GnRH-1 (Fig. 2A-D) or Peripherin and GnRH-1 (Fig. 2I,J) revealed that despite the dramatic tangling of the olfactory and vomeronasal fibers observed in the Arx-1^null^ mutants, the GnRH-1 ns were nonetheless able to bypass the tangle and access the brain (Fig. 2B,D,J). In both controls and Arx-1^null^ mutants, the GnRH-1 ns were seen migrating to the brain along OMP-negative fibers (Fig. 2C,D).

Quantification of total GnRH-1 numbers at E13.5 (WT=715±87; KO=676±38; *n*=3), E15.5 (WT=727±76; KO=725±39; *n*=4) and P0 (WT=576±82; KO=661±99; *n*=3) indicated there were no differences between genotypes at any of the analyzed stages (mean±s.e.m., unpaired *t*-test *P*>0.05). To establish if the GnRH-1 ns migrate in the brain at a different rate in Arx-1 mutants when compared to control animals, we quantified the distributions of GnRH-1 ns between the nasal area and the brain at E13.5, E15.5, and P0 (Fig. 2K). This analysis revealed no difference among genotypes (Fig. 2K).

Even in wild-type mice, a subset of GnRH-1 ns never reach the preoptic/hypothalamic area, but instead remain in the nasal area or form rings around the OBs (Casoni et al., 2016). Quantification of GnRH-1 ns in the olfactory bulb/forebrain junction and brain (Fig. 2L), indicated that both at E15.5 (not shown) and P0, a similar number of GnRH-1 ns remains in the FCM in Arx-1^null^ mutants compared with those found around the OBs in controls (Fig. 2L).

**Fig. 3.**
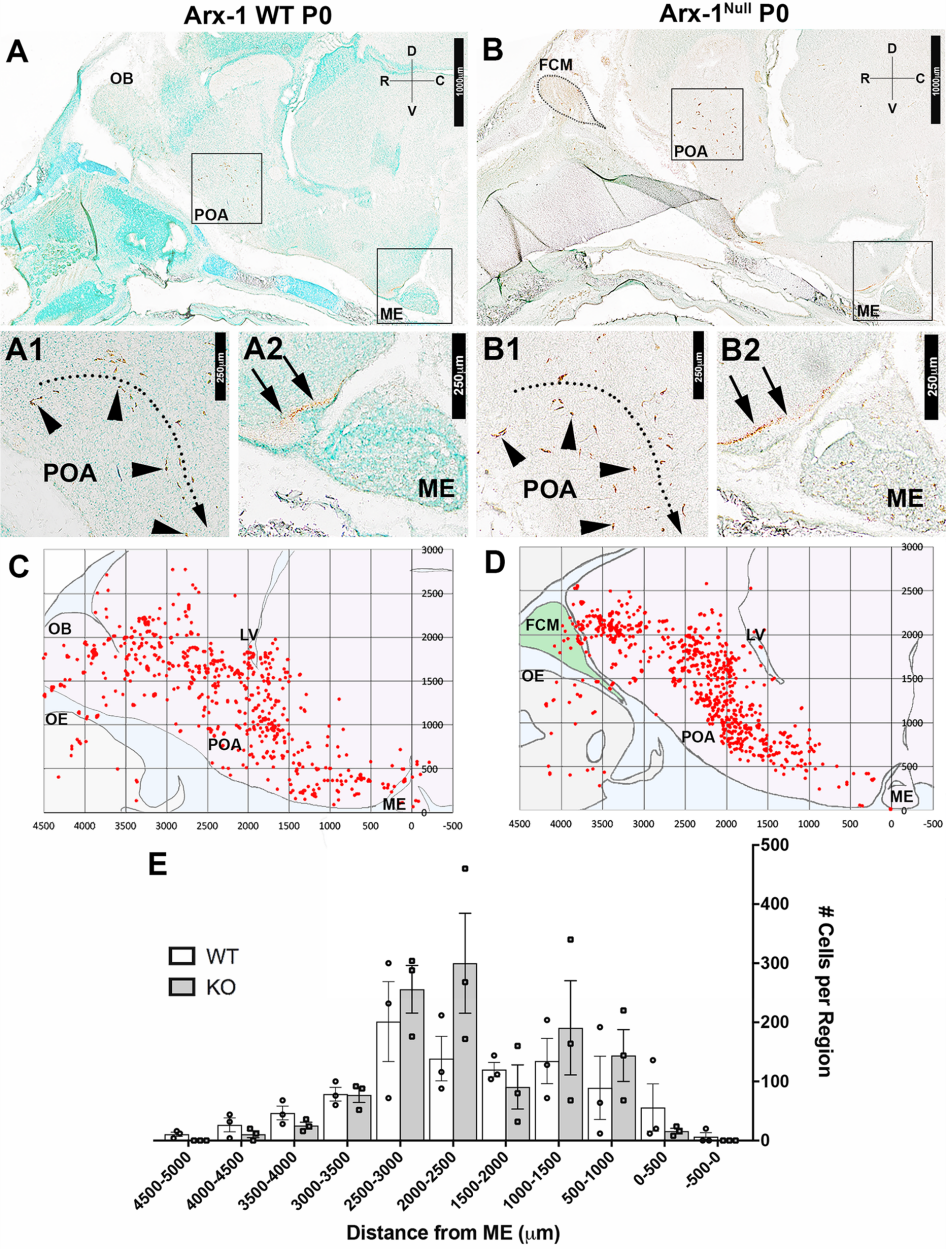
**Aberrant formation of the olfactory system does not significantly affect the rostro-caudal distribution of GnRH-1 ns in the brain.** (A-B2) Immunostaining against GnRH-1 on parasagittal sections on P0 Arx-1 WT (A-A2) and KO (B-B2) show similar distribution. (A1,B1) GnRH-1 ns cell bodies (arrowheads) in the POA and their (A2,B2) projections (arrows) to the ME (D, dorsal; V, ventral; R, rostral; C, caudal). Mapping of the GnRH-1 ns distribution in Arx-1 WT (*n*=3) and null (*n*=3) mice was performed on one series from each animal. (C,D) Scatter plots illustrating GnRH-1 ns distribution in WT (C) (*n*=3) and KO (*n*=3) (D), obtained by overlapping the GnRH-1 ns coordinates from one series from each animal in reference to the median eminence (0,0 µm), in along similar migratory paths. (E) Histogram illustrating the average number of GnRH-1 ns on the rostral-caudal axis in 500 µm intervals. The mean cell counts for the WT from distances –500-0 µm: 6.667± 6.667, 0-500 µm: 56±40.067, 1000-1500 µm: 134.667±38.251, 1500-2000 µm: 120±12.20, 2000-2500 µm: 138.667±37.547, 2500-3000 µm: 201.333±67.580, 3000-3500 µm: 78.667±11.2624, 3500-4000 µm: 46.667± 11.624, 4000-4500 µm: 26.667±11.851, 4500-5000 µm: 10.667±3.528. The mean cell counts for the Arx-1 Null from distances –500-0 µm: 0, 0-500 µm: 16±4.619, 1000-1500 µm: 144±43.879, 1500-2000 µm: 90.667±79.644, 2000-2500 µm: 300±84.664, 2500-3000 µm: 256±40.266, 3000-3500 µm: 77.333±12.719, 3500-4000 µm: 25.333±5.812, 4000-4500 µm: 10.667± 5.812, 4500-5000 µm: 0 (data are mean±s.e.m., unpaired student's *t*-test, *P*>0.05). FCM, fibrocellular mass; LV, lateral ventricle; ME, median eminence; OB, olfactory bulb; POA, pre-optic area.

Performing a detailed mapping of GnRH-1 distribution in control and Arx-1^null^ mutants at P0, we observed comparable distribution of the GnRH-1 ns, from the entry point, ventral to the olfactory bulbs, to the caudal hypothalamus, proximal to the median eminence (Fig. 3) to controls. However, the GnRH-1 cells in the Arx-1 mutants appeared to be more clustered along the dorso-ventral axis when compared to controls (Fig. 3C,D).

Thus, our analyses at these developmental stages argued that during embryonic development the GnRH-1 ns migrate into the forebrain at a comparable rate as in control mice, regardless of the severity of the olfactory bulb aplasia and the dramatic defects in olfactory axonal termination.

### The fibers upon which the GnRH-1 ns access the brain are distinct from the olfactory fibers

**Fig. 4.**
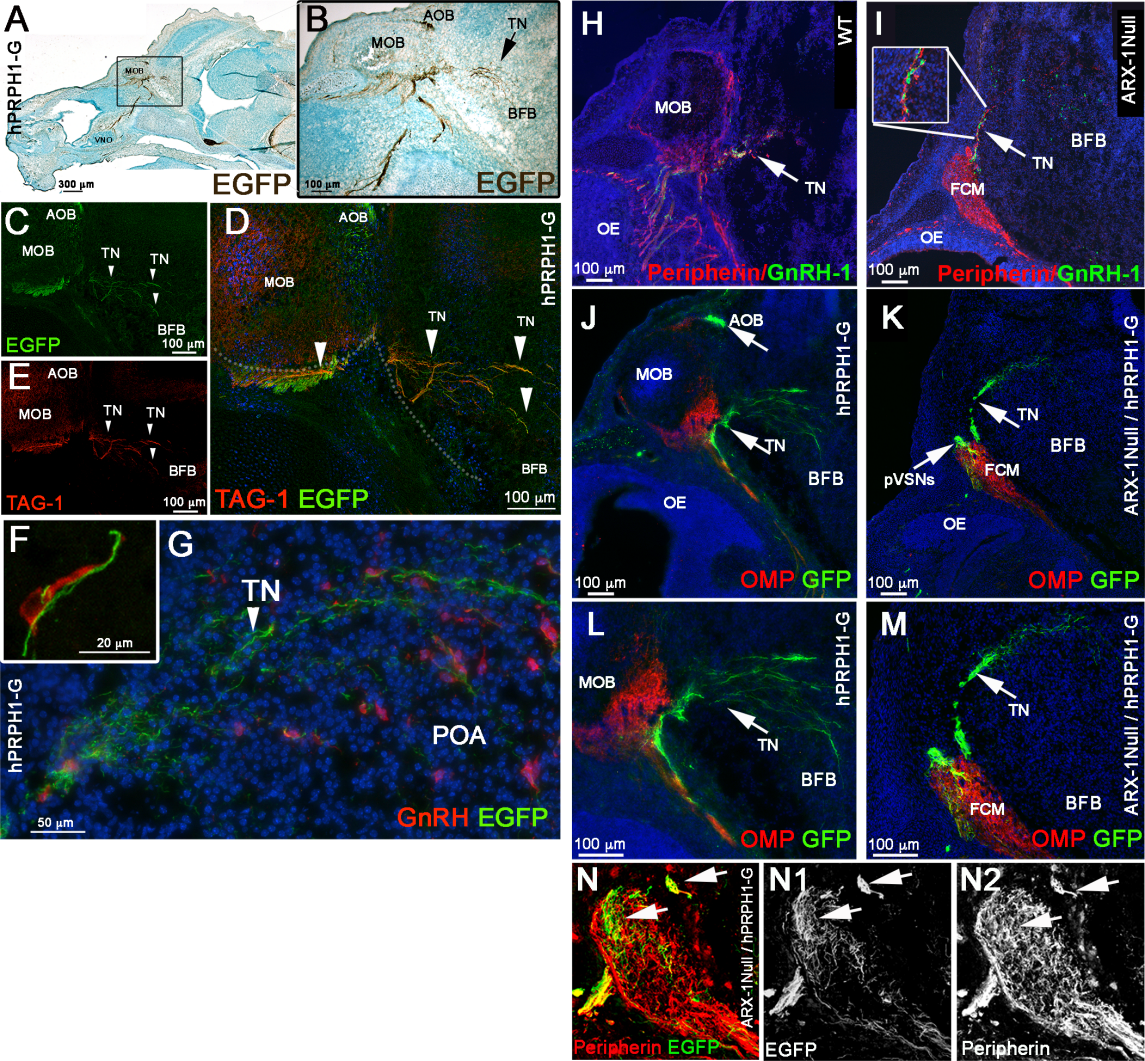
**Expression of EGFP under the control of a human Peripherin promoter distinguishes the TN from the olfactory/vomeronasal nerve.** (A-G) E15.5, parasagittal sections on hPRPH-1G. (A,B) Immunostaining against EGFP showing strong EGFP expression in the AOB and TN invading the basal forebrain (BFB). (C-E) Double immunofluorescence against TAG-1 and EGFP, both EGFP and TAG-1 were found to be strongly expressed in the TN fibers, low TAG-1 expression was detected in putative vomeronasal fibers (VF) projecting to the AOB. (F,G) E15.5, immunofluorescence against EGFP and GnRH-1. GnRH-1 ns (red) access the brain along Peripherin/EGFP-positive TN fibers. (H-O) hPRPH1-G mice reveal that the TN is distinct form OSNs and bypasses the FCM in Arx-1^null^ mutants. (H,I) Peripherin/GnRH-1 double immunostaining on E15.5 WT (H) and Arx-1^null^ (I) GnRH-1 ns enter the forebrain along the TN fibers positive for Peripherin (arrows). (I) In the Arx-1^null^, GnRH-1 ns access the brain along the Peripherin-positive TN fibers that emerge from the Peripherin-positive FCM. (J,L) hPRPH1-G, E15.5, double immunostaining against OMP and EGFP, OMP+ olfactory sensory neurons projecting to the MOB are mainly negative for EGFP expression while the VSNs projecting to the AOB and the TN invading the basal forebrain (BFB) are positive for EGFP (arrows). (K,M) Double immunostaining against OMP and EGFP on hPRPH1-G /Arx1^null^, E15.5. The FCM is composed of OMP+ collapsed axons (red; tangled fibers) mainly negative for EGFP expression while the fibers of the TN, positive for EGFP (arrows), are able to access the brain as in control animals (compare to K,M). Putative VSNs (pVSNs) were found to be tangled as part of the FCM together with the OSNs strongly positive for OMP. (N-N2) E15.5 hPRPH1-G /Arx1^null^. Immunostaining against Peripherin and EGFP shows that hPRPH1-G is selective for pVSNs and the TN (arrows).

Immunostaining for endogenous Peripherin in mice is usually used to highlight axons of cranial nerves, including those of the olfactory/vomeronasal and TN/cranial nerve 0 (Casoni et al., 2016; Schwanzel-Fukuda, 1999; Wray et al., 1994; Yoshida et al., 1995). Thus, one of the major technical limitations in developmental studies of GnRH-1 ns is the lack of molecular markers able to selectively label neuronal subpopulations in the nasal area. While analyzing the *hPRPH1-G* BAC transgenic line, which expresses EGFP under control of a human Peripherin gene promoter (McLenachan et al., 2008), we observed in the nasal area that expression of the hPeripherin:EGFP fusion protein was not fully consistent with that of the endogenous mouse gene. Whereas the endogenous mouse Peripherin protein was readily detectable by immunostaining for Peripherin on OSNs, VSNs and on fibers forming the GnRH-1 migratory pathway (Fig. 4H,I,N), *hPRPH1-*EGFP expression was strong only in putative VSNs projecting to the accessory olfactory bulb (AOB) and in TN neurons and barely detectable in OSNs projecting to the main olfactory bulb (MOB) (Fig. 4A,B,J,L). Immunostaining against TAG-1, which was previously found to highlight neurons forming the GnRH-1 migratory scaffold (Casoni et al., 2016; Yoshida et al., 1995), and GnRH-1 confirmed that the EGFP+ fibers projecting to the basal forebrain were fibers of the presumptive TN (Fig. 4C-G).

To validate that the GnRH- ns follow the same migratory route in Arx-1^null^ mutants and controls, we exploited the stronger selective EGFP expression of the *hPRPH1-G* BAC transgenic line in VN and TN fibers (Fig. 4H-M). In line with what was observed after OMP/GnRH-1 immunolabeling (Fig. 2), immunolabeling against EGFP and OMP on *hPRPH1-G* mutant mouse sections (Fig. 4K,M) indicated that the fibers, upon which the GnRH-1 access the brain either did not express OMP or expressed it below immunodetectable levels. To follow selectively the trajectories of the putative TN in controls and Arx-1^null^ mice we generated *hPRPH1-G*^+/−^/ Arx-1^null^ embryos (Fig. 4K,M,N). Observations on these embryos revealed that the TN projections accessing the brain were positive for hPRP1-G expression, as was seen in control animals (Fig. 4J,L) while the hPRP1-G expressing vomeronasal sensory axons were tangled as part of the FCM (Fig. 4K,M,N).

### The TN fibers are distinct from apical and basal vomeronasal sensory neurons

**Fig. 5.**
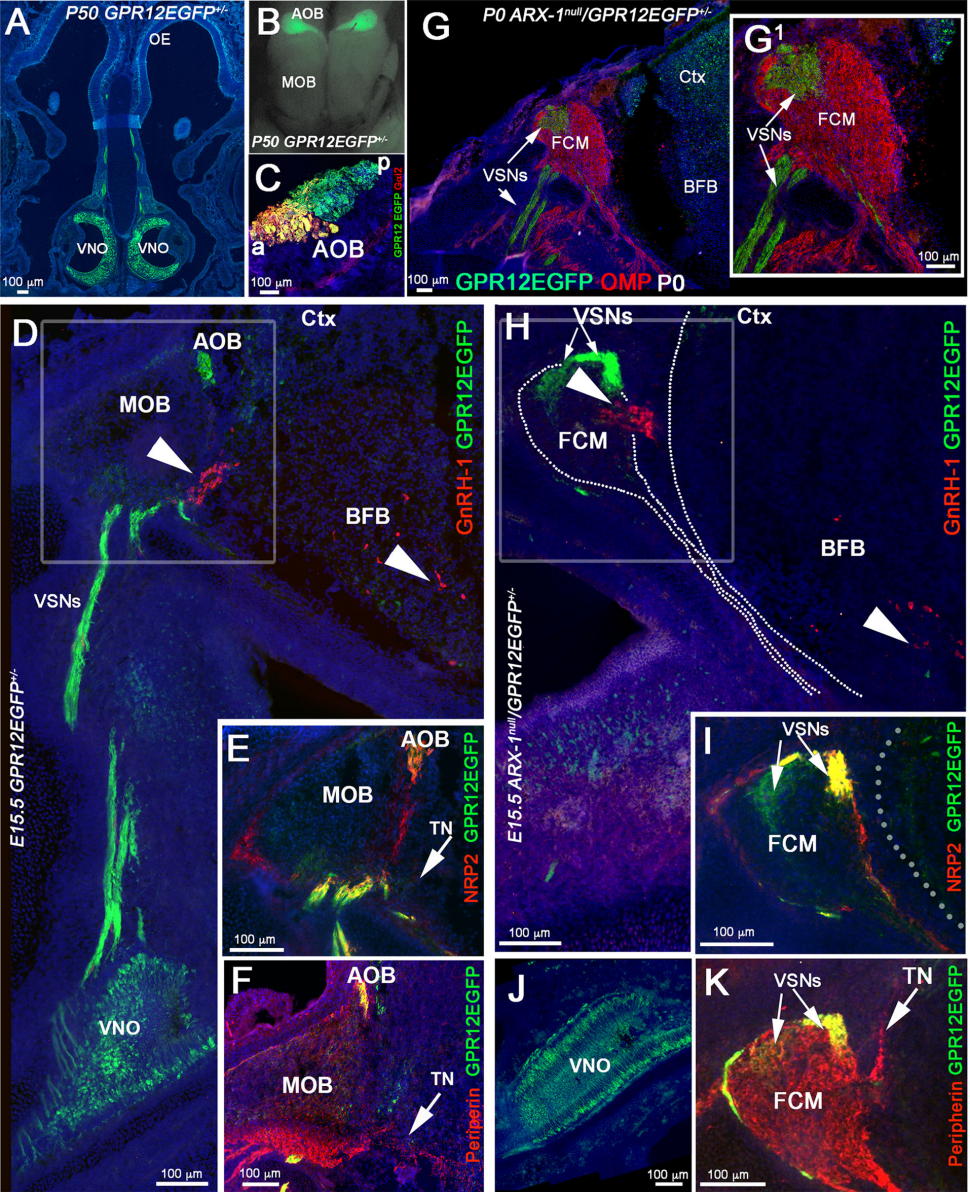
**GPR12-EGFP BAC transgenics show that the TN is distinct from VSNs.** (A-C) Postnatal GPR12-EGFP. (A) Coronal section; EGFP expression is limited to the VSNs and to sparse cells in the OE. (B) Whole mount; EGFP is detectable in VSNs projecting to the AOB but not in those projecting to the MOB. (C) Gαi2/EGFP staining on parasagittal section of the AOB, showing that the EGFP-positive fibers project to both the anterior (a) and posterior (p) AOB. (D-F) E15.5 GPR12-EGFP. (D) Double immunostaining against GnRH-1 and EGFP. The GnRH-1 ns access the brain along GPR12-EGFP-negative fibers (arrowheads) while GPR12-EGFP+ axons project from the VNO to the AOB. (E) NRP2 (red) /EGFP (green) double staining showing NRP2 in GPR12-EGFP+ positive axonal bundles projecting to the AOB and in OSNs projecting to the MOB. (F) Peripherin/EGFP double immunofluorescence; EGFP is expressed in the VSNs projecting to the AOB but not in the Peripherin+ TN (arrow). (G,G1) P0, Arx-1^null^/GPR12-EGFP immunostaining against OMP (red) and EGFP (green). The GPR12-EGFP-positive vomeronasal fibers (VSNs) project toward the brain and collapse as part of the FCM. (H-K) E15.5 Arx-1^null^/GPR12-EGFP. (H) GnRH-1 (red) accessing the brain square (arrowhead in the BFB) on GPR12-EGFP-negative fibers; the EGFP+ VSNs collapse as part of the FCM. (I) NRP2 /EGFP double staining shows NRP2 expression in OSNs and in GPR12-EGFP+ VSNs in the FCM. (J) EGFP expression in the VNO of E15.5 Arx-1^null^/GPR12-EGFP (K) Peripherin/EGFP double immunofluorescence; Peripherin highlights the FCM and the TN emerging from the FCM, while EGFP is expressed by the VSNs but not by the TN (arrows).

Whereas some earlier researchers had proposed that GnRH-1 ns reach the hypothalamus on a set of vomeronasal (VN) fibers that diverge from those that project to the accessory olfactory bulb (AOB) (Wray et al., 1989a; Yoshida et al., 1995), others argued instead that GnRH-1 ns migrate along axons of the elusive TN, which initially forms bundles with the vomeronasal axons until they diverge (Casoni et al., 2016; Schwanzel-Fukuda and Pfaff, 1989; Vilensky, 2012; Zhao et al., 2013). To resolve this discrepancy, we searched for genetic markers that could distinguish among the various subpopulations of axons resident in the nasal regions where GnRH-1 ns migrate. The vomeronasal organ (VNO) itself is composed of apical and basal subpopulations of sensory neurons (VSNs). Both subpopulations project to the AOB, but they differ in their final targets, expression of adhesion molecules, Semaphorin receptors (Neuropilins), Slit receptors (Robos), VN receptors, G-protein subunits, and glycoproteins (Francia et al., 2014; Perez-Gomez et al., 2014; Schwarting et al., 1994). The apical neurons project to the anterior (a) portion of the AOB, whereas the basal neurons project to the posterior (p) AOB. The neuronal branch upon which GnRH-1 ns migrate shares transient expression of glycoproteins expressed by the basal, but not the apical VSNs (Yoshida et al., 1995). By analyzing microarray data obtained from vomeronasal tissue, data (not shown), we found that the G-protein coupled receptor GPR12, which is a high-affinity receptor for sphingosylphosphorylcholine (Ignatov et al., 2003), is expressed in the developing VNO (data not shown, available at www.eurexpress.org). We next tested whether an EGFP-tagged version of GPR12 could be used to selectively label the vomeronasal axons, using the GENSAT GPR12-EGFP BAC transgenic mouse line. Analysis of these mice (Fig. 5) indicated that indeed the vomeronasal neurons, along with a few, sparse olfactory and microvillar neurons in the main olfactory epithelium (data not shown), expressed GPR12 from embryonic to postnatal stages. Immunostaining with two markers that can distinguish apical from basal VSNs, Gαo (not shown) and Gαi2 (Chamero et al., 2012), revealed that GPR12-EGFP was expressed in both the apical and basal VSNs, which respectively project to the anterior (a) and posterior (p) portions of the AOB (Fig. 5A-C). By immunostaining sections from these mice at E15.5 (*n*=3; Fig. 5D-F) for GnRH-1, Peripherin, and NRP2, we found that fibers of the TN were genetically distinct from these axons. Moreover, migrating GnRH-1 ns were found to deviate from the GPR12-EGFP+ vomeronasal bundles projecting to the AOB (Fig. 5D) and to access the brain along Peripherin-positive fibers negative for GPR12-EGFP, consistent with the presence of distinct TN fibers (Fig. 5F). Furthermore, the GPR12-EGFP expression allowed us to confirm that NRP2 expression was limited to the olfactory and apical VSN axons (Fig. 5E). In addition to the GnRH-1 ns, we also observed sporadic migratory cells expressing GPR12 in their cell body migrating together with the GnRH-1 ns (data not shown).

By mating Arx-1^null^ females with GPR12-EGFP males, we generated Arx-1^null^/GPR12-EGFP^+/−^ mice and GPR12-EGFP^+/−^ controls. Using these mice, we could selectively follow the trajectory of developing vomeronasal sensory fibers in the absence of proper olfactory bulb development by examining embryos at E15.5 and at birth (P0). In the Arx-1 mutants at P0 (Fig. 5G,G1), the VSNs axons formed a tangle within the FCM, surrounded by olfactory fibers. Analysis of Arx-1^null^/GPR12-EGFP^+/−^ embryos at E15.5 by immunostaining for EGFP in combination with GnRH-1, Peripherin, NRP2 (Fig. 5H-K) confirmed that the GnRH-1 ns crossed the FCM and accessed the brain along neurons negative for GPR12EGFP. Collectively, these data showed that the axons of the TN differ from vomeronasal axons, and are used by GnRH-1 ns to access the brain.

### The GnRH-1 ns and TN differ from the vomeronasal fibers for guidance receptors expression

**Fig. 6.**
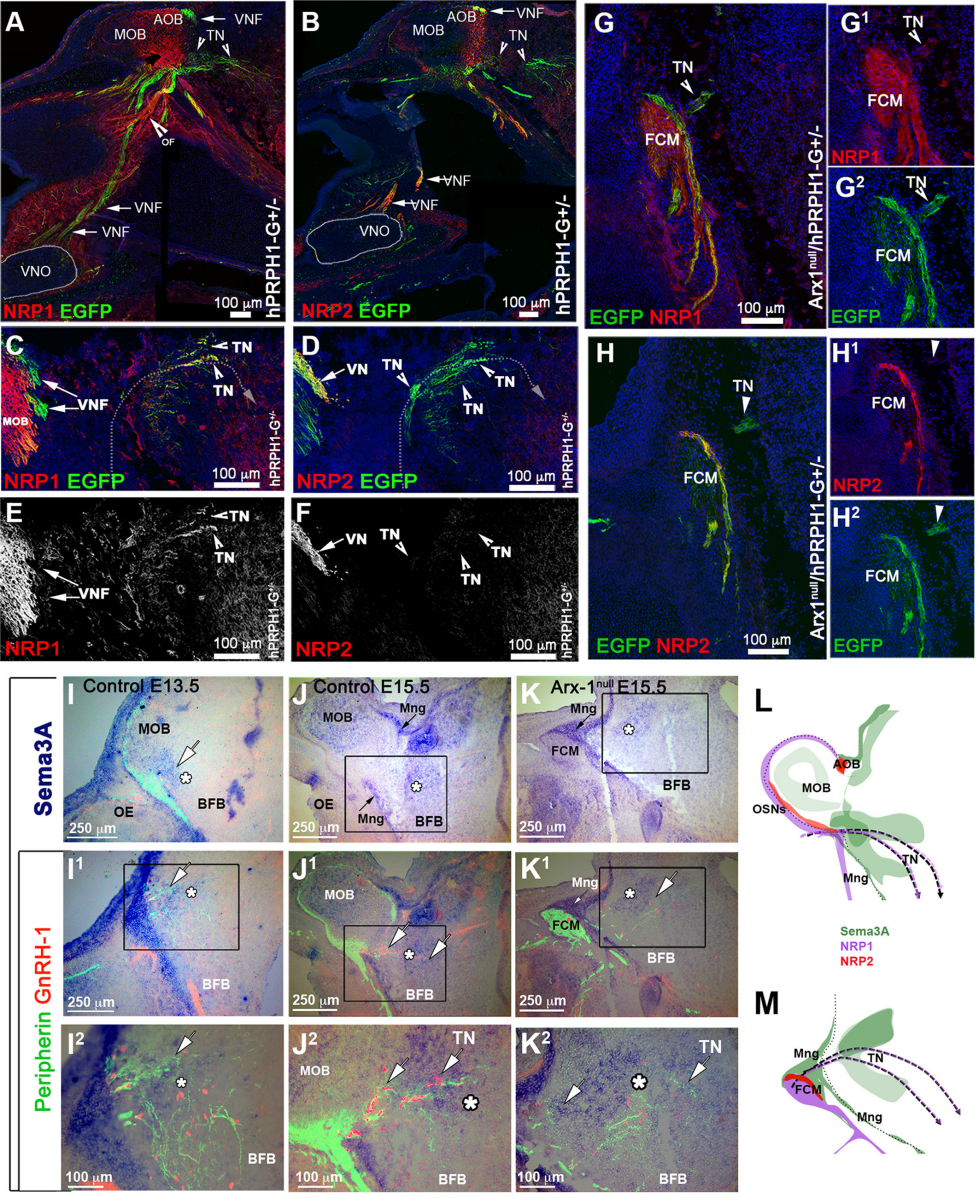
**GnRH-1 ns and the TN invade the brain proximal to a source of Sema3A.** (A,C,E) hPRPH1G^+/−^ E15.5; immunostaining against NRP1 and EGFP. (A) EGFP is strongly expressed by pVSNs projecting from the VNO to the AOB and by pTN fibers accessing the brain ventral to the MOB. NRP1 immunoreactivity was not found along vomeronasal fibers (VNF) but in the nasal mesenchyme on the fibers of the OSNs neurons (OF) projecting to the MOB (yellow arrow). (C,E) Enlargements showing the TN fibers accessing the brain express low levels of NRP1. (B,D,F) hPRPH1G^+/−^ E15.5; immunostaining against NRP2 and EGFP. NRP2 was strongly expressed the axonal fibers of the VSNs (VNF) by subsets of fibers of the OF projecting to the MOB (arrows). (D,F) Enlargements showing the TN fibers accessing the brain are negative or below detectability for NRP2 (notched arrows). (G-H2) Arx1^null^/hPRPH1-G^+/−^ E15.5. (G-G2) Immunostaining against NRP1 and EGFP reveals that while NRP1+ olfactory fibers are repelled from the developing telencephalon and collapse as part of the FCM the fibers of the TN, positive for EGFP (notched arrowheads), branch out of the FCM and project towards the brain. (H-H2) IF against EGFP and NRP2; putative VN fibers positive for NRP2 and EGFP were found to be part of the FCM while the fibers of the pTN branch out of the FCM and project towards the brain. (I-K2) ISH against Sema3A and IF against Peripherin and GnRH-1 on E13.5 (I-I2) and E15.5 (J-K2) controls (I-J2) and Arx-1^null^ (J), showing Sema3A expression in the forebrain (asterisks). In both controls and Arx-1^null^ mutants, strong Sema3A expression was found on the meninges (Mng, arrows) around the brain. In Arx-1^null^ mutants strong Sema3A was expressed on the meninges in contact with the FCM. (K-K2) IF against Peripherin and GnRH-1 on ISH against Sema3A reveals that the GnRH-1 ns and the TN enter the brain in correspondence and in close proximity to a large source of Sema3a in Arx-1^null^ and controls. (I2, J2, K2) Enlargements showing the TN fibers and GnRH-1 ns accessing the brain through regions of Sema3a. (L,M) Schematics summarizing the trajectories of the olfactory, vomeronasal and TN (dotted line) with respect to NRP1, NRP2 expression and sources of Sema3A in the brain and meninges.

The key regulators of olfactory axonal routing and targeting are the Class-3 Semaphorins, Neuropilin receptors (NRP-1 and NRP-2), Slit1, Slit2, and Roundabout (Robo) receptors (Cho et al., 2012; Renzi et al., 2000; Schwarting et al., 2000; Takeuchi et al., 2010; Walz et al., 2002). Slit and Sema3 proteins prevent olfactory fibers from invading the brain prior to OB formation (Renzi et al., 2000). Immunostaining against the Sema receptors NRP-1 and NRP-2 on hPRPH1-G^+/−^ mice showed that during GnRH-1 neuronal migration, NRP1 and NRP2 were strongly expressed by olfactory sensory neurons, projecting to the main olfactory (MOB) and by vomeronasal sensory neurons projecting to the accessory olfactory bulb, respectively (Fig. 6A-F). However, TN fibers, which are strongly EGFP+ in these mice, exhibited only weak NRP1 expression and no detectable NRP2 expression (Fig. 6A-F). Similarly, in Arx-1^null^/hPRPH1-G double mutants, we observed that NRP1 and NRP2 were expressed by the olfactory and vomeronasal fibers in the FCM, whereas the EGFP+ fibers of the TN expressed NRP1 only weakly, and no detectable NRP2 (Fig. 6G-H2).

To further understand the relationship between the aberrant olfactory/vomeronasal trajectories and successful GnRH-1 migration in Arx-1^null^ mutants, we performed *in situ* hybridization (ISH) against the diffusible guidance cues Semaphorin 3A (Sema3A). By combining this digoxigenin-based ISH with double immunofluorescence for Peripherin and GnRH-1, we could follow the TN trajectory with respect to this guidance cue in the brain.

Analysis of WT animals at E13.5 and E15.5 consistently showed the TN and the GnRH-1 ns invade the brain ventral and between the developing OBs in a region positive for Sema3A expression (Fig. 6I1-K2).

Consistent with observations in WT, analysis of Arx-1^null^ mutants at E15.5 showed that the FCM, which is mainly formed by NRP1+ fibers (Fig. 6G-G2) collapsed in close proximity with meninges positive for Sema3A expression (Fig. 6K,K1). However, the GnRH-1 ns and TN were found to be able to penetrate the brain and to project towards and across sources of Sema3A (Fig. 6K1-2).

### The GnRH-1 ns and TN respond differently from the olfactory and vomeronasal fibers to sources of Slit1 in the brain

**Fig. 7.**
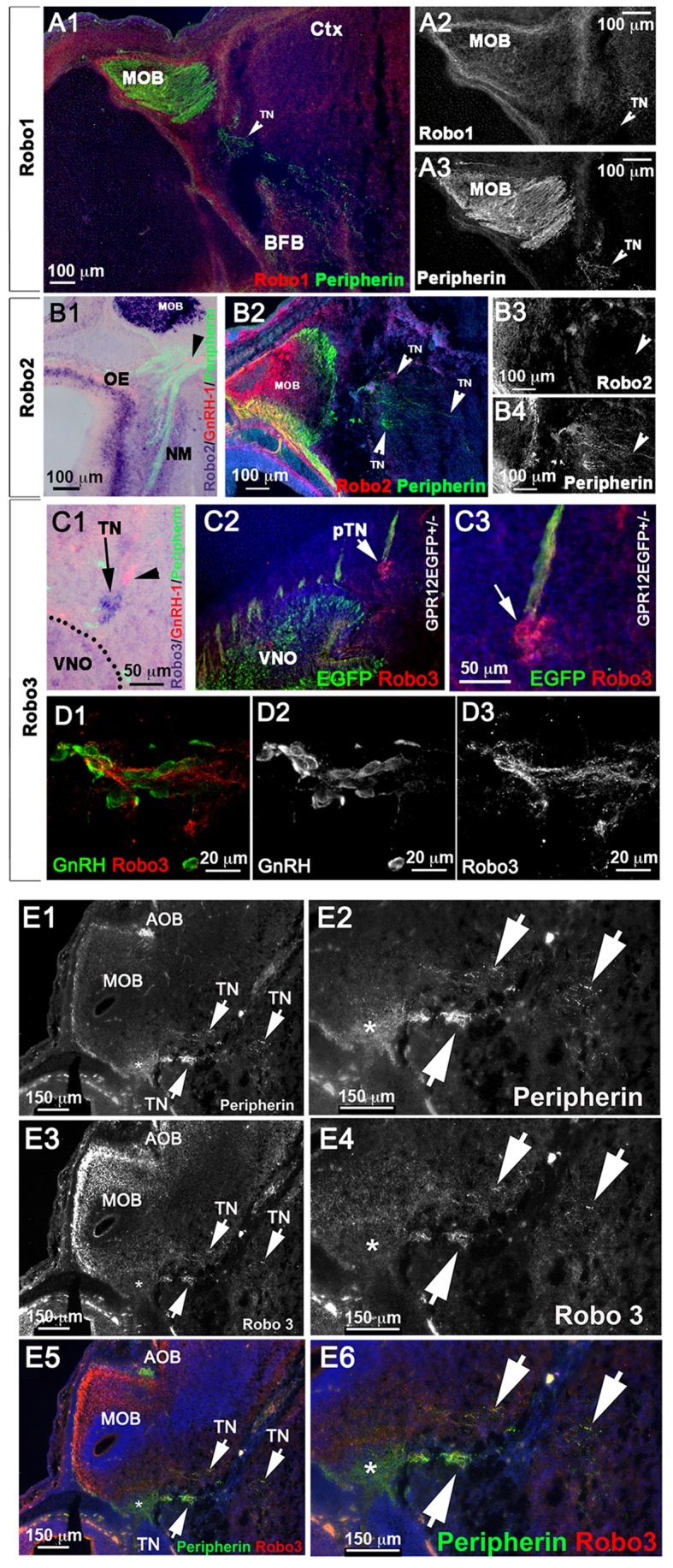
**The TN is positive for Robo3 but not for Robo1 or Robo2.** (A1-B4) E15.5 WT animal. (A1-3) Double immunostaining against Robo1 and Peripherin shows that the Peripherin+ TN is negative for Robo1. (B1) ISH against Robo2 combined with immunofluorescence against Peripherin and GnRH-1. Robo2 was detected in the vomeronasal neurons, olfactory epithelium (OE), nasal mesenchyme (NM) and in the olfactory bulb. No Robo2 was detected in GnRH-1 ns. (B2-B4) Immunofluorescence against Robo2 and Peripherin shows lack of immunoreactivity for Robo2 in the TN accessing the brain. (C1) E15.5 WT animal, ISH against Robo3 combined with IF against GnRH-1 and Peripherin. Strong Robo3 mRNA expression was found in cells proximal to the VNO negative for GnRH-1 immunoreactivity (arrow). (C2,C3) E15.5 GPR12-EGFP immunostained for EGFP and Robo3 confirms Robo3 expression in cell bodies and fibers of neurons proximal to the VNO forming bundles with GPR12-EGFP+ VSNs (arrows in C2 and C3). Robo3+ cells proximal to the VNO negative for GnRH-1 and EGFP are indicated as pTN. (D1-D3) E15.5 WT animal. GnRH-1 and Robo3 double immunofluorescence reveals migrating GnRH-1 ns in contact with Robo3+ fibers. (E1-E6) Double immunofluorescence against Robo3 and Peripherin in WT animals (E15.5). The fibers of the pTN accessing the brain are positive for Robo3 and Peripherin immunoreactivity.

**Fig. 8.**
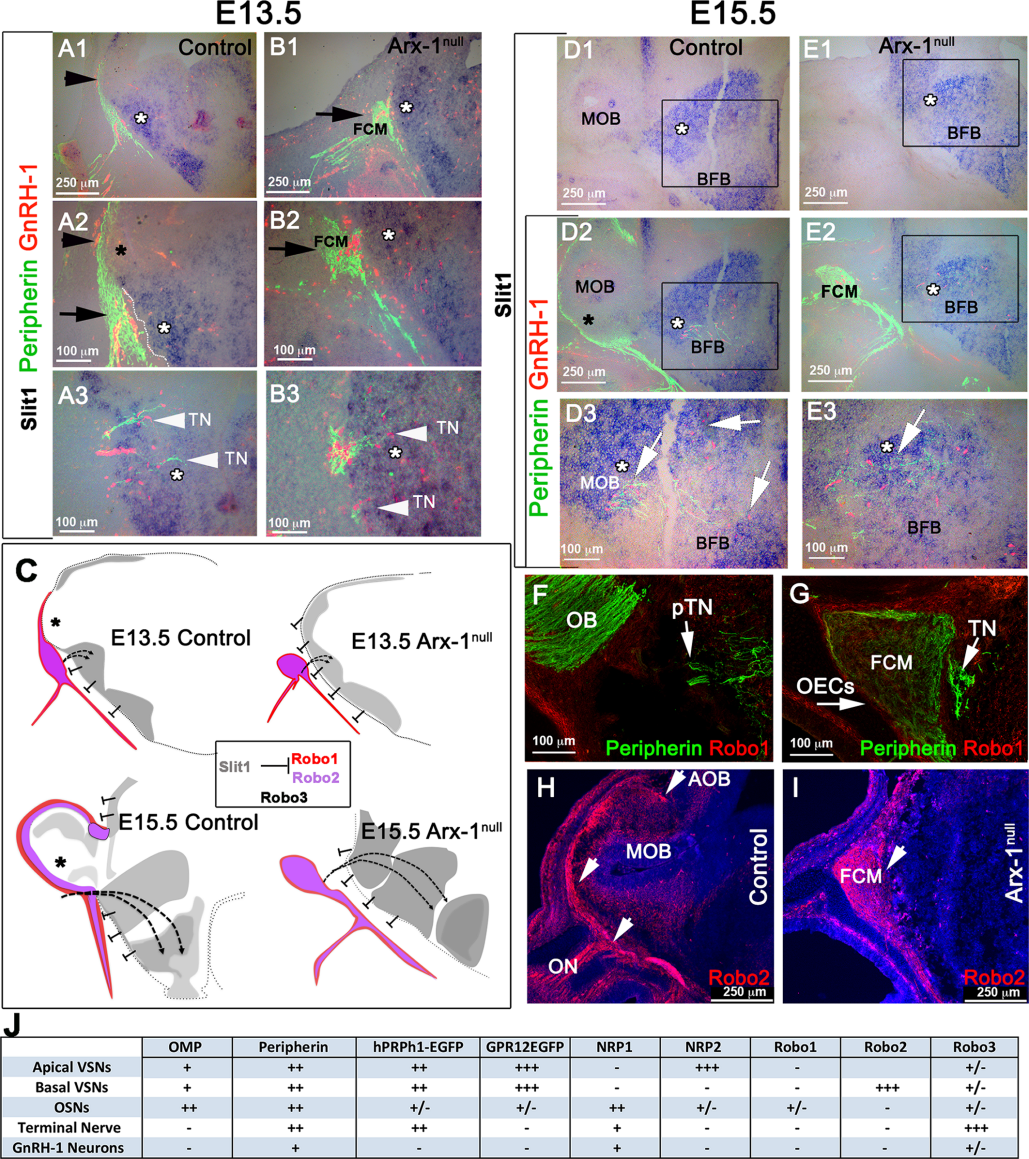
**TN and GnRH-1 ns invade the brain in areas of Slit1 expression.** (A-B3) ISH against Slit1 (blue) combined with immunofluorescence against Peripherin and GnRH-1 in E13.5 WT (A1-A3) and Arx-1^null^ mutants (B1-B3). (A1,A2) Slit1 is expressed in the cortex and basal forebrain (white asterisks); however, the Peripherin-positive ON and VSNs (black arrowheads) contact the brain in the area negative for Slit1 (black asterisk) where the OB will form. (A3) GnRH-1 ns (red) invade the brain along Peripherin-positive TN crossing a large source of Slit1. (B1-B3) In the Arx-1^Null^, the entire rostral border of the brain expresses Slit-1. The olfactory and vomeronasal fibers did not access the brain and instead collapse, forming the FCM (black arrows) facing areas of Slit1 expression (white arrowheads). The GnRH-1 ns (red) cross the FCM and penetrate the brain (white arrowheads) in Slit1 expression (white asterisks) areas as in controls. (C) Model illustrating the relationship between Robo1, Robo2, Robo3 and Slit1 in controls and Arx-1^null^ mutants during development. The TN trajectories have been indicated with dashed lines. Slit-free areas are indicated by black asterisks. (D1-E3) ISH against Slit1 (blue) combined with immunofluorescence against Peripherin and GnRH-1 in E15.5 WT (D-D3) and Arx-1^null^ mutants (E1-E3). Slit1 was found expressed (white asterisks) in the cortex and basal forebrain. In control animals, the olfactory and VSNs Peripherin+ fibers were found to project to the OB, which is mainly negative for Slit1 (black asterisk) while in the KOs, the olfactory and vomeronasal fibers did not access the brain strongly positive for Slit1. In both controls (D2,D3) and Arx-1^null^ (E2,E3) GnRH-1 ns and Peripherin-positive TN fibers (white arrows) access the brain crossing large sources of Slit1 (white asterisks). (F) WT E15.5; Robo1/Peripherin immunofluorescence shows lack of Robo1 expression in the TN accessing the brain (arrows). (G) E15.5 Arx-1^null^; Robo1 immunofluorescence was detected on the olfactory ensheathing cells (OECS) surrounding and within the FCM (arrowhead), no Robo1 immunoreactivity was found in the TN (arrow). (H) WT E15.5; immunofluorescence anti Robo2 shows expression in the olfactory fiber projections to the MOB and VSNs projections to the posterior AOB. (I) Arx-1^null^ (E15.5); Robo2 immunofluorescence shows Robo2 expression in the axons of the FCM facing the source of Slit1 (D2). (J) Summary of the molecular differences found between olfactory, vomeronasal, GnRH-1 and neurons of the pTN.

Slit proteins play a pivotal role in repelling Robo1+ and Robo2+ olfactory and vomeronasal neurons and in preventing them from invading the brain (Nguyen-Ba-Charvet et al., 2008; Renzi et al., 2000). Consistent with this report, we observed, by ISH at E15.5, that both the olfactory and vomeronasal neurons expressed Robo2 (Fig. 7B1). Similarly, immunofluorescence confirmed detectable Robo2 expression in axons of the developing OSNs as well as in subsets of VSN projecting to the OB and tangled in the FCM of the Arx-1^Null^ mutants (Fig. 8H,I). Also, Robo1 expression was detected in putative olfactory ensheathing cells (OECs) (Aoki et al., 2013) surrounding the olfactory bulb in controls and surrounding the FCM in Arx-1^null^ mutants (Fig. 7A1-A3 and Fig. 8F,G). Consistent with a repellant role for Slit proteins in repelling Robo1+ and Robo2+ axons, ISH for Slit1 on controls and Arx-1^null^ mutants revealed strong Slit1 expression in the cortex as well as in the basal forebrain (Fig. 8A1-E3).

In sharp contrast to the OSNs and VSNs, neither the GnRH-1 ns nor the TN expressed detectable levels of either Robo1 (Fig. 7A1-A3 and Fig. 8F,G) or Robo2 (Fig. 7B2-B4).

A third member of the Robo gene family of receptors, Robo3, does not bind Slit proteins, but various isoforms of Robo3 can silence Slit-mediated repulsion if they are co-expressed with Robo1 or Robo2 (Sabatier et al., 2004; Zelina et al., 2014). ISH and immunohistochemistry against Robo3 revealed barely detectable levels of Robo3 expression in olfactory and vomeronasal neurons, but axons of the TN were strongly positive for Robo3 protein expression (Fig. 7E3,E4). Furthermore, Robo3 mRNA was strongly expressed in cell bodies forming a ganglionic structure proximal to the VNO in the nasal area (Fig. 7C1). Robo3 immunostaining of GPR12-EGFP embryos confirmed that this staining was not in VSN cell bodies (Fig. 7C2,C3). In the nasal region, Robo3 fibers comingled with GPR12-EGFP+ VSN fibers, and Peripherin immunostaining confirmed strong immunoreactivity for Robo3 on the fibers of the putative TN (Fig. 7C2,C3,E1-E6). Migrating GnRH-1 ns were found along Robo3-expressing axons (Fig. 7D1-D3).

To follow the trajectories of the OSN and VSNs with respect to sources of Slit in the brain at E13.5 and E15.5, we coupled ISH against Slit1 with Peripherin and GnRH-1 immunofluorescence. At both stages, the OSN/VSNs contacted the brain in areas negative for Slit1 expression, which correspond to the putative olfactory bulb primordia (Fig. 8A1,A2,C,D2). However, in Arx-1^null^ mutants, where the OBs fail to properly form, Slit1 was expressed in a continuum throughout the cortex and basal forebrain (Fig. 8B1,B2,C,E1,E2). Whereas the OSNs and VSNs positive for Robo1 and Robo2 (Fig. 8F-I) collapse proximal to sources of Slit1 (Nguyen-Ba-Charvet et al., 2008) (Fig. 8B1-B3,C,E2), the TN and GnRH-1 ns appeared to be able to invade the brain and to migrate across sources of Slit1 (Fig. 8A3,B3,D3,E3).

Collectively, these results (see summary in Fig. 8C,J) suggest that GnRH-1 ns migrate from the nose to the brain along axons of the TN. Our experiments suggest that routing of the axonal projections of the TN is defined by signaling mechanisms distinct from those controlling olfactory and vomeronasal targeting to the olfactory bulbs.

## DISCUSSION

After the initial description of KS (Kallmann et al., 1944) and subsequent discovery that GnRH-1 ns migrate from the nose to the brain (Schwanzel-Fukuda and Pfaff, 1989; Wray et al., 1989a,b), a link between olfactory development and GnRH-1 migration was proposed, investigated and accepted (Cariboni et al., 2007; Lewkowitz-Shpuntoff et al., 2012; Toba et al., 2008; Wray, 2010).

However, the incomplete penetrance of anosmia and HH in families carrying KS (de Roux, 2005; Karstensen and Tommerup, 2012; Moya-Plana et al., 2013; Pitteloud et al., 2005; Trarbach et al., 2006) led us to question whether this link was truly causal. We thus analyzed GnRH-1 development in the Arx-1^null^ model, where the loss of the Arx-1 gene by precursors of OB interneurons severely compromises olfactory bulb development without compromising the olfactory placode and its derivatives. The normally developing telencephalon releases repulsive cues to prevent the penetration of olfactory fibers, thereby directing them to the OBs (Cloutier et al., 2002; Nguyen-Ba-Charvet et al., 2008; Renzi et al., 2000). Thus, in Arx-1^null^ mutants as in other mouse models of arhinencephaly, the absence or reduction of OBs forces the olfactory and vomeronasal sensory fibers to form axonal tangles where the OBs should be (Balmer and LaMantia, 2004; Imai et al., 2009).

Despite the brain defects, aberrant OB formation, and the extreme misrouting of olfactory/vomeronasal axons, the migratory rate of the GnRH-1 ns, as well as their ability to reach the preoptic/hypothalamic areas was not obviously altered in Arx-1 mutants. Arx-1 loss affects normal development of the brain (Friocourt et al., 2006; Simonet et al., 2015); therefore, some differences in how cells scatter in the brain could reflect abnormalities in the brain parenchyma (Fig. 3). These results indicate that targeting of olfactory and vomeronasal axons to the OB does not play a fundamental role in defining the rate, and routing of GnRH-1 ns migration into the forebrain. Instead, GnRH-1 ns appeared to migrate along the axons of the putative TN to access the brain.

By exploiting hPRPH-1G and GPR12-EGFP BAC transgenics we revealed a distinction between the putative TN and the olfactory and vomeronasal sensory neurons. The GPR12-EGFP BAC transgenic was found to be expressed by VSNs, few olfactory neurons, but not by the TN neurons. Though we cannot exclude that some of the cell bodies of the TN might be within the developing VNO, our data point to a distinct identity for this nerve from the VSNs (Yoshida et al., 1995).

A small number of GnRH-1 ns was found to fail to enter the brain in the FCM of Arx-1^null^ mutants. However, even in normal animals, a similar number of GnRH-1 ns migrate on fibers projecting to the OB (Casoni et al., 2016). This suggests the existence of a subpopulation of GnRH-1 ns that invariably migrates to the OB along specific neurons that must differ from the majority that project to the hypothalamus. If the GnRH-1 ns that migrate to the OBs (Casoni et al., 2016) play active roles in olfaction is a possibility that should be further investigated.

Strengthening the idea that the TN and not the olfactory/vomeronasal fibers provides the scaffold for GnRH-1 ns migration, we showed that these different subpopulations of axons follow different guidance cues. Targeting of axons in general is defined by a complex interplay of attractive and repulsive signals (Brignall and Cloutier, 2015; Cho et al., 2012, 2007; Cloutier et al., 2002, 2004; Prince et al., 2009; Schwarting et al., 2000). An array of GnRH-1 migratory defects, with varying severity, occurs in genetically modified animal models, in which axonal misrouting and/or defasciculation of olfactory neurons also occurs (Barraud et al., 2013; Cariboni et al., 2015, 2012, 2011; Hernandez-Miranda et al., 2011; Matsumoto et al., 2006; Messina et al., 2011; Pingault et al., 2013; Tillo et al., 2015). However, discriminating between cell-autonomous and secondary effects of mutations of the genes linked to KS in humans (e.g. prokineticin-2, prokineticin receptor-2, Fgf8, Fgf8-Receptor-1, Semaphorin3A, Semaphorin7A, Sox10 and CHD7) is made difficult by the broad number of tissue/cell types affected (Hanchate et al., 2012; Lewkowitz-Shpuntoff et al., 2012). Semaphorin 3A, NRP1, NRP2, Slit proteins and the receptor Robo3 have all been previously implicated in guiding olfactory axons and GnRH-1 ns (Cariboni et al., 2012, 2011).

By performing ISH against Sema3A and Slit1 on control and Arx-1^null^ mice we confirmed that the forebrain is a large source of repellent molecules, which, in concert, might prevent the olfactory and vomeronasal fibers, but not the TN nor the GnRH-1 ns, from invading the brain (Nguyen-Ba-Charvet et al., 2008; Renzi et al., 2000). We observed that the Sema3 receptors, NRP1 and NRP2, are expressed by olfactory and vomeronasal neurons projecting to different regions of the MOB and AOB (Cloutier et al., 2002, 2004; Taku et al., 2016; Walz et al., 2002); however, we could only detect NRP1 but not NRP2 immuno-reactivity on GnRH-1 ns and TN axons (Cariboni et al., 2011; Giacobini, 2015; Giacobini and Prevot, 2013; Hanchate et al., 2012). By performing ISH against Sema3A in combination with immunofluorescence for GnRH-1 and Peripherin, we observed that the TN and GnRH-1 invade the brain crossing a source of Sema3A.

Loss of Sema3A expression compromises both olfactory/vomeronasal axon trajectories as well as those of the TN, along with GnRH-1 ns migration. The effects of class 3 Semaphorins on growth cone trajectories varies according to Sema3 concentration, NRP expression levels, and levels of cyclic nucleotides, which can selectively favor growth cone attraction versus repulsion (Manns et al., 2012; Pond et al., 2002; Song et al., 1998). In Arx-1 mutants, NRP1+ and NRP2+ olfactory and VSNs axons collapsed proximal to the brain as part of the FCM (Fig. 6), whereas in both wild-type and Arx-1^null^ mutants, the TN projected towards a source of Sema3A. These observations, together with the phenotype of Sema3A KO, where TN and GnRH-1 fail to invade the brain (Cariboni et al., 2011; Giacobini and Prevot, 2013), suggest that Sema3A may prevent olfactory and vomeronasal fibers from invading the brain while attracting GnRH-1 ns and TN fibers.

Robo1 and Robo2 receptors cause axonal collapse in response to Slit proteins, whereas Robo3, depending on the isoform, can silence Slit repulsion (Chen et al., 2008). In line with previous studies, we found that both olfactory neurons and subsets of vomeronasal neurons express Robo2, along with low levels of Robo1 and Robo3. In mice lacking Robo1/Robo2, the olfactory fibers invade the forebrain, following a route similar to that followed by the TN (Nguyen-Ba-Charvet et al., 2008). However, despite significant olfactory defects, Robo1 and Robo2 double mutants have no GnRH-1 migratory defects (Cariboni et al., 2012). Also, our data on WT and Arx-1^null^ mutants showed that the cortex and basal forebrain are large sources of Slit1, and that the GnRH-1 ns, in contrast to the olfactory and vomeronasal axons, cross sources of Slit proteins in the forebrain (Fig. 8). In line with these observations, TN and GnRH-1 ns (data not shown) are negative for Robo1 and Robo2 expression, but positive for Robo3. A previous study described defects in GnRH-1 migration in Robo3^null^ animals, which was proposed to be in response to Robo3 binding Slit2 (Cariboni et al., 2012) and would seem to contradict our findings. However, Robo3 is now known to bind NELL2 and not Slit proteins (Camurri et al., 2005; Jaworski et al., 2015; Zelina et al., 2014). Therefore, defective GnRH-1 migration in Robo3 mutants could result from the inability of TN and GnRH-1 ns to respond to NELL2 mediated guidance.

Our conclusion that the TN, and not the olfactory/vomeronasal sensory neurons, provides the scaffold for GnRH-1 ns migration, is supported by comparative phylogenetic studies. For example, although the VNO is absent or vestigial in birds, amphibians, and fish and cetaceans and humans (Bang, 1971; Dulac and Torello, 2003; Smith and Bhatnagar, 2000; Zancanaro, 2014), GnRH-1 ns and the TN connecting the nose have been described in these species (Demski and Schwanzel-Fukuda, 1987; Fuller and Burger, 1990; Mousley et al., 2006; Muske and Moore, 1988; Ridgway et al., 1987; von Bartheld et al., 1987; Zhao et al., 2013).

Our work has shown, for the first time, that the invasion of the brain by migrating GnRH-1 ns is independent from the correct targeting of the olfactory and vomeronasal neurons to the OBs. Although an impaired sense of smell, absence or reduction of olfactory bulb volume are all common diagnostic parameters for KS, our work suggests that neither defects in olfactory bulb development nor aberrant olfactory/vomeronasal axonal routing are sufficient to prevent the migration of GnRH-1 ns into the basal forebrain. The pathophysiological overlap between KS and normosmic IHH in humans (Lewkowitz-Shpuntoff et al., 2012) implies, that although the development of the TN/GnRH-1 system and the olfactory system may rely on partially overlapping guidance cues, they follow different molecular mechanisms. To reach a full understanding of the molecular mechanisms leading to KS and normosmic IHH, the community should now attempt to isolate and fully characterize the cells of the TN nerve in different animal systems and humans.

## MATERIALS AND METHODS

### Animals

Cryopreserved Arx-1^null^ mice were resuscitated from the Riken repository. The origins of these mice and their olfactory defects were previously described (Kitamura et al., 2002; Yoshihara et al., 2005), and our Arx-1 colony was C57 BL/6J mixed background. Because the *Arx-1* gene is located on the X-chromosome, *Arx-1* hemizygous null mutants were also genotyped for sex to identify male mutants (see below). Arx-1^null^ mice do not survive past postnatal day 0 (P0); therefore, animal cages were checked early in the morning on the day of birth. Embryos of different stages were collected after euthanizing time-mated dams. Peripherin-EGFP (hPRPH1-G) mice were obtained from Dr J. Sasero (Murdoch Childrens Research Institute, Melbourne, Australia) on a C57BL/6J background (McLenachan et al., 2008). hPRPH1-G males were mated with Arx-1 females to generate hPRPH1-G /Arx-1^null^ embryos. GPR12EGFP BAC transgenic mice were resuscitated from GENSAT repository at MMRRC, UC Davis. These mice were obtained on a mixed background. Because EGFP expression in the VNO varied among the F_0-_resuscitated animals obtained from GENSAT, we selected a subline (GPR12EGFP^395^) with persistent strong expression in apical and basal vomeronasal sensory neurons. GPR12EGFP^395^ male mice were mated with Arx-1 females to generate Arx-1^null^/GPR12EGFP embryos. Animals were euthanized using CO_2_, followed by cervical dislocation. All animal procedures were in accordance with procedures approved by the University at Albany Institutional Animal Care and Use Committee (IACUC).

### Tissue preparation

Embryos and heads were collected from time-mated *Arx-1* females at E13.5, E15.5 and P0, and the emergence of the copulation plug was taken as E0.5. Collected embryos were immersion-fixed in 3.7% Formaldehyde/PBS at 4°C for 3 h. P0 heads were immersion-fixed in the same fixative at 4°C for overnight. All samples were then cryoprotected in 30% sucrose overnight or until they sank, then frozen in OCT (Tissue-TeK) using dry ice, and kept at −80°C. Samples were cryosectioned using a CM3050S cryostat (Leica, Wetzlar, Germany) and collected on Superfrost plus slides (VWR, Radnor, PA, USA) at 12-16 μm for immunostaining and 18-25 µm for ISH.

### Confirmation of animal genotypes

The genotypes of the mice were established by polymerase chain reaction (PCR) analysis using the following primers: *Arx-1^null^* (mArx fff: 5′-CGCCC AAGGA AGAGC TGTTG CTGC-3′; *ARX pMC1neo stop*: 5′-GCCTT CTTGA CGAGT TCTTC-3′; ARX mArx eer 5′TATTC CACCC TCCTG GACCC TTTC-3′); *EGFP* (eGFP fwd: 5′-CCTAC GGCGT GCAGT GCTTC AGC-3′; *eGFP REV:* 5'CGGCG AGCTG CACGC TGCGT CCTC-3′); *Actin 470* (*ACTIN SENSE*): 5′-CTCGT CTGGGA AAGCA GAAAC TGCAA-3′; *ACTIN 470* (*ANTISENSE*): 5'-GTGAC CTGTT ACTGG GAGTG GCAAG C-3′). Amplification products were analyzed by agarose gel electrophoresis.

### Immunohistochemistry

Primary antibodies and dilutions used were as follows: goat-α-neuropilin-1(1:400, R&D Systems, Minneapolis, MN, USA), goat-α-neuropilin-2 (1:3000, R&D Systems), goat-α-ROBO1 (1:200, R&D Systems), goat-α-ROBO3 (1:50, R&D Systems), mouse-α-ROBO2 (1:50, Santa Cruz Biotechnology Headquarters Dallas, TX, USA) chicken-α-peripherin (1:1500, Abcam), rabbit-α-peripherin (1:2000, Millipore), SW rabbit-α-GnRH-1 (1:6000, Susan Wray, NIH), rabbit-α tyrosine hydroxylase (1:1000, Abcam), goat-α olfactory marker protein (1:4000, WAKO), goat-α transient-axonal glycoprotein 1 (1:1000, R&D Systems), rabbit-α-GFP (1:2000, Molecular Probes, Eugene, OR, USA), chicken-α-GFP (1:1000, Abcam), mouse-α-GAD67 (1:200, Santa Cruz Biotechnology). Antigen retrieval was performed in a citric acid solution prior to incubation with chicken-α-peripherin, rabbit-α-tyrosine hydroxylase, mouse-α-ROBO2, and α-GAD67 antibodies. For immunoperoxidase staining procedures, slides were processed using standard protocols (Forni et al., 2013) and staining was visualized (Vectastain ABC Kit, Vector, Cambridgeshire, UK) using diaminobenzidine (DAB) in a glucose solution containing glucose oxidase to generate hydrogen peroxide; sections were counterstained with methyl green. For immunofluorescence, species-appropriate secondary antibodies were conjugated with Alexa Fluor 488, Alexa Fluor 594, or Alexa Fluor 568 (Molecular Probes and Jackson Laboratories) as specified in the legends. Sections were counterstained with 4′,6′-diamidino-2-phenylindole (1:3000; Sigma-Aldrich) and coverslips were mounted with Fluoro Gel (Electron Microscopy Sciences, Hatfield, PA, USA). Confocal microscopy pictures were taken on a LSM 710 microscope (Zeiss, Oberkochen, Germany). Epifluorescence pictures were taken on a DM4000 B LED fluorescence microscope equipped with a Leica DFC310 FX camera. Images were further analyzed using Fiji/ImageJ software (http://fiji.sc/#download, Schindelin et al., 2012). Each staining was replicated on at least three different animals for each genotype. See Table S1 for full details of the primary and secondary antibodies used in this study.

### X-gal staining

Sections were rehydrated in PBS and then incubated in a solution of 5 mM potassium ferrocyanide, 5 mM potassium ferricyanide, 2 mM MgCl_2_, 0.1% Tween, and 0.1% 5-bromo-4-chloro-3-indolyl-b-D-galactoside/dimethylformamide at 37°C ON After the enzymatic reaction was completed, slides were either counterstained with 1% Eosin-Y (Electron Microscopy Services) or washed and immunostained as described above.

### ISH

Digoxigenin-labeled cRNA probes were prepared by *in vitro* transcription (DIG RNA labeling kit; Roche Diagnostics, Basel, Switzerland) from the following templates: Semaphorins 3A, (Kagoshima and Ito, 2001); Slits-1, as well as Robo-2, Robo-3 (Cloutier et al., 2004). ISH was performed as described (Forni et al., 2013) and visualized by immunostaining with an alkaline phosphatase conjugated anti-DIG (1:1000), and NBT/BCIP developer solution (Roche Diagnostics). Sections were then counter-immunostained with antibodies against both chicken-α-peripherin, and SW rabbit-α-GnRH-1, as described above for immunofluorescence.

### Mapping the distribution of GnRH-1 ns

Whole heads of P0 Arx-1^null^ mutants and controls (*n*=3;3) were cryosectioned at 16 µm thickness. The sections were then immunostained against GnRH-1 in DAB and counterstained with methyl green. The most medial 16 sections (eight sections from either side of the midline cartilage) were scanned at 10× using a VS120 Olympus scanning microscope in brightfield. Sections were aligned in PhotoShop CS6 using the median eminence, cerebellum, and ventricles as landmarks. Cell bodies were marked and overlaid, representing a cross section of their migratory path. The coordinates of each cell body were plotted in reference to the origin (x=0;y=0), which was set at the middle of the median eminence, using FIJI. The number of GnRH-1 ns distributed along the x-axis (rostro-caudal) were quantified in 500 µm intervals for each animal. Differences at each interval between genotypes was assessed by unpaired *t*-test.

### Experimental design and statistical analyses

All statistical analyses were carried out using GraphPad Prism7 software. Cell counts were performed on serial sections immunostained for GnRH-1 at E13.5 (*n*=3), E15.5 (*n*=4) and P0 (*n*=3), and visualized under bright field (immunoperoxidase) or epi-fluorescence illumination (20×; Leica DM4000 B LED), according to their anatomical location [i.e. (1) nasal region (VNO, axonal tracks surrounding the olfactory pits, forebrain junction); (2) olfactory bulb/fibrocellular mass; and (3) brain (all the cells that accessed the olfactory bulb and were distributed within the forebrain)]. For each animal, counts were performed on three serial series. The average number of cells from these three series was then multiplied by the total number of series/animal to compute a value for each animal. These were then averaged to obtain the mean±standard error of the mean (s.e.m.) among animals of the same age and genotype. Means±s.e.m. were calculated from at least three animals per genotype. The statistical differences between genotypes and groups were determined using unpaired student's *t*-test. All data are presented as the mean±s.e.m. from *n*≥3 mice per genotype/age for each experiment. *P*<0.05 was considered to be statistically significant.
